# Extraction, purification, structural elucidation, and immunomodulatory activity of *Bletilla striata* polysaccharides via the NF-κB signaling pathway

**DOI:** 10.3389/fimmu.2026.1749545

**Published:** 2026-01-28

**Authors:** Guofeng Pang, Kai Zhou, Di Gao, Danli Wang, Juan Xue, Tian Lu, Huifang Chai

**Affiliations:** College of Pharmacy, Guizhou University of Traditional Chinese Medicine, Guiyang, China

**Keywords:** *Bletilla striata*, immunomodulatory activity, NF-κB signaling pathway, polysaccharide, RAW 264.7 macrophages

## Abstract

**Introduction:**

*Bletilla striata* is a traditional medicinal plant, and its polysaccharide components have been confirmed to possess immunomodulatory potential. However, most current studies focus on high-molecular-weight polysaccharides, while the immunomodulatory mechanism of low-molecular-weight polysaccharides from this plant remains unclear. This study aimed to isolate and identify a low-molecular-weight polysaccharide (BSP-2) from *B. striata* tubers, and to explore its immunomodulatory activity as well as the related action mechanism.

**Methods:**

A neutral polysaccharide (BSP-2) was isolated from *B. striata* tubers. Its molecular weights were characterized using high-performance gel permeation chromatography (HPGPC) and nuclear magnetic resonance (NMR); its monosaccharide composition was determined via component analysis. The immunomodulatory activity of BSP-2 was evaluated using lipopolysaccharide (LPS)-stimulated RAW264.7 cells (by detecting pro-inflammatory mediator production), and its mechanism was investigated through the analysis of relevant protein expressions.

**Results:**

BSP-2 exhibited the following molecular weight parameters: number-average (3.612 ± 0.13646 kDa), weight-average (4.456 ± 0.18019 kDa), z-average (6.364 ± 0.5518 kDa), and peaked molecular weight (2.808 ± 0.09041 kDa). Component analysis revealed that BSP-2 was dominated by glucose (Glc), with minor proportions of galactose (Gal), arabinose (Ara), and rhamnose (Rha) residues. In LPS-stimulated RAW264.7 cells, BSP-2 inhibited the production of four pro-inflammatory mediators: IFN-γ, TNF-α, IL-1β, and IL-6. Mechanistically, the anti-inflammatory effects of BSP-2 were mediated by the regulation of the NF-κβ pathway, which involved the downregulation of proteins including p65, p-p65, IκBα, p-IκBα, COX-2, and iNOS.

**Discussion:**

These results demonstrate that BSP-2 balances pro-inflammatory responses via the NF-κB pathway, supporting its promising application as a bioactive immunoregulatory component in functional food and dietary supplement development.

## 1 Introduction

*Bletilla striata* (Thunb.) Reichb.f., a terrestrial orchid of the genus Bletilla, is renowned for its hemostatic, anti-inflammatory, tissue-regenerative, and wound-healing properties (1). Recognized in China as a traditional medicine-food homology resource, it exhibits low toxicity and is considered a safe natural food ingredient. *B. striata* is utilized in brewing and medicinal cuisine due to its distinctive aroma and good palatability (2). Moreover, composite preservatives formulated from *B. striata* have been shown to delay mango decay and significantly extend the postharvest shelf life of mangoes (3). *B. striata* is rich in bioactive compounds, including polysaccharides, flavonoids, saponins, phenanthrenes, and bibenzyls. Among these, *B. striata* polysaccharide (BSPs) is considered a primary bioactive constituent, exhibiting excellent film-forming ability and strong antioxidant activity. Notably, a composite coating prepared from paeonol and BSP has been reported to effectively extend the shelf life of grapes by maintaining the activity of superoxide dismutase (SOD) on the fruit surface and forming a protective film (4). Additionally, due to its favorable safety profile and inherent viscosity, BSP is widely employed as a food ingredient or additive (2), demonstrating promising application potential in the food industry.

Studies have shown that *Bletilla striata* extract can effectively suppress the secretion of inflammatory cytokines such as interleukin-1β (IL-1β), transforming growth factor-β (TGF-β), interleukin-6 (IL-6), and tumor necrosis factor-α (TNF-α) by downregulating the expression of c-Jun N-terminal kinase (JNK) and p38 in the MAPK signaling pathway, thereby significantly inhibiting MAPK pathway activation. The anti-inflammatory activity is further supported by the inhibition of p65 phosphorylation (p-p65) in the NF-κB pathway (5). Mannose (Man) and glucose (Glc) are the major components of *B. striata* polysaccharide (6). This polysaccharide alleviates LPS- or CTX-induced inflammatory injury by simultaneously blocking both NF-κB and MAPK signaling pathways, leading to reduced production of IL-4, IL-6, IL-1β, and TNF-α (7, 8). Additionally, *B. striata* polysaccharide exhibits various pharmacological effects, including anti-fibrotic (9), antioxidant (10), and antibacterial (11) activities.

Current research on *Bletilla striata* polysaccharides (BSPs) has predominantly focused on high-molecular-weight fractions, particularly those obtained via hot-water extraction, which typically range in molecular weight from 282.91 to 402.17 kDa (12). In contrast, structural elucidation and activity mechanism studies of low-molecular-weight oligosaccharides remain relatively scarce, representing a significant research gap that warrants further investigation.

In this study, we employed, for the first time, a combination of DEAE-650M anion-exchange chromatography and Sepharose 6B gel-filtration chromatography to purify BSP. The purified fraction, designated BSP-2, underwent systematic structural characterization. Subsequently, its potential anti-inflammatory and immunomodulatory effects were evaluated using an LPS-induced RAW 264.7 macrophage model.

These findings provide important theoretical support for the potential application of BSP-2 in functional foods, such as anti-inflammatory health beverages or additives. Furthermore, this work establishes a foundation for developing standardized food ingredients based on specific polysaccharide structures.

## 2 Materials and methods

### 2.1 Materials

The medicinal material of *Bletilla striata* was sourced from a cultivation base in Shibing County, Qiandongnan Prefecture, Guizhou Province, and was botanically identified as *Bletilla striata*.

DEAE-650M anion-exchange column and Sepharose 6B gel-filtration column were obtained from GE Healthcare. Cell Counting Kit-8 (CCK-8), dialysis tubing, ELISA kits for interferon-gamma (IFN-γ), TNF-α, IL-1β, and IL-6, as well as dexamethasone, were purchased from Solarbio (Beijing, China).

Phospho-p65 (Ser536) (39H1) rabbit monoclonal antibody, IκBα (4D4) rabbit monoclonal antibody, phospho-IκBα (Ser32) (14D4) rabbit monoclonal antibody, inducible nitric oxide synthase (iNOS) antibody, and cyclooxygenase-2 (COX-2) antibody were acquired from Cell Signaling Technology (CST, USA). Actin antibody was obtained from HUABIO (China).

RAW 264.7 macrophages were supplied by Shanghai Fuheng Biological Technology Co., Ltd. (China). Analytical-grade reagents were provided by ANPEL Laboratory Technologies (Shanghai) Inc. (China). Manuscript Formatting.

### 2.2 Preparation of BSP-2

Dried *Bletilla striata* tubers were ground and passed through a 40-mesh sieve. The powder was extracted with 14 volumes of distilled water at 68 °C for 94 min. The extract was filtered successively through gauze and then fast-filter paper. This extraction process was repeated three times. The combined filtrates were concentrated to a suitable volume, and absolute ethanol was added to achieve a final ethanol concentration of 85 %. The resulting precipitate was collected, freeze-dried, redissolved in distilled water, and dialyzed for 48 h using dialysis tubing with a molecular weight cut-off of 7 kDa. After dialysis, the solution was centrifuged at 4000 r/min for 20 min. The supernatant was collected, concentrated, and freeze-dried to obtain crude *Bletilla striata* polysaccharide (BSP).

BSP was redissolved in distilled water and centrifuged at 4000 rpm for 15 min. The supernatant was loaded onto a DEAE-650M anion-exchange column and eluted sequentially with distilled water, 0.05 M, 0.3 M, and 0.5 M NaCl solutions to preliminarily separate neutral polysaccharides from acidic polysaccharides and to remove impurities such as proteins and pigments from the crude polysaccharide. The eluates were collected, concentrated, and freeze-dried to obtain the BSP-1 fraction.

BSP-1 was redissolved in distilled water and dialyzed for 48 h using dialysis tubing with a molecular weight cut-off of 7 kDa. After dialysis, the solution was centrifuged at 4000 r/min for 20 min. The supernatant was concentrated and further purified by Sepharose 6B gel-filtration chromatography (eluted with water). Based on differences in polysaccharide molecular weight, this step removed residual heteropolysaccharides of varying molecular weights that were not fully separated in the previous step, yielding a more homogeneous polysaccharide fraction. The eluate was concentrated and freeze-dried to obtain the BSP-2 fraction.

### 2.3 Molecular weight and homogeneity analysis of BSP-2

BSP-2 samples were dissolved in DMSO at a final concentration of 1 mg/mL and filtered through a 0.45 μm membrane filter prior to analysis. The chromatographic system consisted of a gel permeation chromatography–refractive index–multi-angle laser light scattering (GPC-RI-MALS) setup. The liquid chromatography system was a U3000 (Thermo, USA). A refractive index detector (Optilab T-rEX, Wyatt Technology, CA, USA) and a multi-angle laser light scattering detector (DAWN HELEOS II, Wyatt Technology, CA, USA) were used for detection. Separation was performed on a series of gel-filtration columns: Ohpak SB-805 HQ (300 × 8 mm), Ohpak SB-804 HQ (300 × 8 mm), and Ohpak SB-803 HQ (300 × 8 mm) connected in series. The column temperature was maintained at 60 °C. The injection volume was 100 μL. The mobile phase consisted of 0.5 % LiBr in DMSO, delivered at a flow rate of 0.3 mL/min under isocratic elution for 120 min. The refractive index detector provided concentration information based on refractive intensity, while the MALS detector collected light-scattering data of macromolecules. The molecular weight of each fraction was calculated using the Mark–Houwink equation (13–16).

### 2.4 Chemical component analysis

The total sugar content of BSP-2 was determined using the phenol-sulfuric acid method. A series of standard glucose solutions was prepared at concentrations of 0, 10, 20, 30, 40, 50, 60, 80, and 100 μg/mL. An aliquot of each standard or sample solution was mixed with phenol and concentrated sulfuric acid, allowed to react, and the absorbance was measured at 490 nm. A standard curve was plotted from the absorbance values of the standard solutions. An appropriate amount of BSP-2 was dissolved and diluted with distilled water, and its absorbance was measured following the same procedure. The total sugar content was calculated based on the standard curve.

Protein content of BSP-2 was quantified using the Bradford method with Coomassie Brilliant Blue G-250. A series of bovine serum albumin (BSA) standard solutions was prepared at concentrations of 0, 0.2, 0.4, 0.6, 0.8, and 1.0 mg/mL. For the assay, 0.1 mL of each standard or sample solution was mixed with 5 mL of Coomassie Brilliant Blue G-250 reagent, vortexed, and incubated at room temperature for 2 min. Absorbance was then measured at 595 nm, and a standard curve was constructed. An appropriate amount of BSP-2 was dissolved and diluted with distilled water, and its absorbance was determined using the same protocol. The protein content was subsequently calculated from the standard curve.

### 2.5 Compositional analysis of monosaccharides

Monosaccharide composition analysis was performed using a Thermo ICS 5000+ ion chromatography system (Thermo Fisher Scientific, USA) equipped with an electrochemical detector. Separation was carried out on a Dionex™ CarboPac™ PA20 column (150 × 3.0 mm, 10 μm) maintained at 30 °C. The injection volume was 5 μL. The mobile phase consisted of three components: A (H_2_O), B (0.1 M NaOH), and C (0.1 M NaOH, 0.2 M NaAc), delivered at a flow rate of 0.5 mL/min. The following monosaccharide standards were used: mannose (Man), glucose (Glc), rhamnose (Rha), xylose (Xyl), galactose (Gal), arabinose (Ara), fucose (Fuc), fructose (Fru), ribose (Rib), galacturonic acid (Gal-UA), glucuronic acid (Glc-UA), mannuronic acid (Man-UA), and guluronic acid (Gul-UA).

For sample preparation, an appropriate amount of BSP-2 was hydrolyzed with 1 mL of 2 M trifluoroacetic acid (TFA) at 121 °C for 2 h. The hydrolysate was dried under a stream of nitrogen. The residue was washed with methanol (99.99 %) and dried again; this methanol washing step was repeated 2–3 times. The final product was dissolved in sterile distilled water and transferred to a chromatography vial for analysis. The monosaccharide composition of the sample was identified by comparing its chromatographic profile with those of the authentic standards. The proportion of each monosaccharide was quantified based on the corresponding standard curve (17).

### 2.6 Methylation analysis

GC-MS analysis was performed using an Agilent 7890A gas chromatography system (Agilent Technologies, USA) equipped with a BPX70 capillary column (30 m × 0.25 mm × 0.25 µm, SGE, Australia). The injection volume was 1 µL with a split ratio of 10:1, using high-purity helium as the carrier gas. The oven temperature was initially held at 140 °C for 2.0 min, then increased to 230 °C at a rate of 3 °C/min, and held for 3 min. Detection was carried out using an Agilent 5977B quadrupole mass spectrometer (Agilent Technologies, USA) equipped with an electron ionization (EI) source and controlled by MassHunter software. Analysis was performed in full scan (SCAN) mode with a mass range of m/z 50–350.

For derivatization, 2 mg of the sample was dissolved in 500 µL of DMSO. Then, 1 mg of NaOH was added, and the mixture was incubated for 30 min. Subsequently, 50 µL of methyl iodide solution was added, and the reaction proceeded for 1 h. Next, 1 mL of water and 2 mL of dichloromethane were added, mixed thoroughly, and centrifuged. The aqueous phase was discarded. This water-washing step was repeated three times. The lower dichloromethane phase was collected and dried under a stream of nitrogen.

Subsequently, 100 µL of 2 M TFA was added, and the mixture was heated at 121 °C for 90 min. After drying at 30 °C, 50 µL of 2 M ammonia and 50 µL of 1 M sodium borodeuteride (NaBD_4_) were added, mixed, and allowed to react at room temperature for 2.5 h. The reaction was terminated by adding 20 µL of acetic acid, and the mixture was dried under nitrogen. The residue was washed twice with 250 µL of methanol and dried again. Then, 250 µL of acetic anhydride was added, mixed, and the reaction was carried out at 100 °C for 2.5 h.

After adding 1 mL of water and letting it stand for 10 min, 500 µL of dichloromethane was added, mixed, and centrifuged. The aqueous phase was discarded, and the water-washing step was repeated three times. The final dichloromethane phase was collected for GC-MS analysis. The positions and proportions of glycosidic linkages in BSP-2 were estimated based on characteristic fragment ions identified by gas chromatography (18, 19).

### 2.7 NMR analysis

NMR analysis was conducted on a Bruker AVANCE NEO 600 MHz spectrometer (Bruker, Germany) at a probe temperature of 25 °C. A QXI 1H/31P/13C/15N 5 mm quadruple-resonance inverse detection probe (Z-gradient, ATM Acc) was used with the following specifications: 1H signal-to-noise ratio (SNR): 888; resolution: 0.32 Hz (rotating). Additionally, a BBFO 1H-19F, 31P-15N, 1H decoupling/observe broadband observe probe (Z-gradient, ATM) was employed with the following specifications: ^1^H SNR: 798; resolution: 0.26 Hz (rotating); ^13^C SNR: 328; resolution: 0.1 Hz.

An appropriate amount of BSP-2 was dissolved in D_2_O to prepare a 40 mg/mL polysaccharide solution. A 0.5 mL aliquot was transferred to an NMR tube for analysis. The following spectra were acquired: one-dimensional ¹H and ¹³C NMR spectra, and two-dimensional COSY, HSQC, NOESY, and HMBC spectra.

### 2.8 Cell viability assay

The complete cell culture medium was prepared by supplementing high-glucose DMEM with 10% fetal bovine serum (FBS) and 1% penicillin-streptomycin. RAW 264.7 macrophages in the logarithmic growth phase were seeded into 96-well plates at a density of 1 × 10^5^ cells/mL, with 100 μL of cell suspension per well. After incubation at 37 °C under 5% CO_2_ for 24 h, the cells were treated with BSP and BSP-2 at various concentrations (0, 7.8, 15.6, 31.3, 62.5, 125, 250, 500, and 1000 μg/mL). Control wells included a blank (medium only, no cells or drug) and a negative control (cells with medium only, no drug). Each experimental condition was performed in triplicate. Following the manufacturer’s instructions for the CCK-8 assay, the absorbance of each well was measured at 450 nm.

### 2.9 Cytokine secretion assay

RAW 264.7 macrophages in the logarithmic growth phase were seeded into 96-well plates at a density of 1 × 10^6^ cells/mL and incubated for 24 h at 37 °C under 5% CO_2_. The experimental groups were established as follows: blank control (medium only), model control (LPS-treated), positive control (0.1 μg/mL dexamethasone), BSP-treated groups, and BSP-2-treated groups (15.6, 62.5, and 500 μg/mL). Each group was set up in triplicate. The blank control received 200 μL of medium. The model control received 198 μL of medium. The positive control received 180 μL of medium and 20 μL of dexamethasone working solution. The high-, medium-, and low-dose groups for both BSP and BSP-2 received 180 μL of medium and 20 μL of the corresponding BSP or BSP-2 working solution.

After pre-incubation for 1.5 h under the same conditions, all groups except the blank control were stimulated with 2 μL of LPS and incubated for an additional 24 h. The plates were then centrifuged at 1000 rpm for 10 min. The supernatants were collected, and the levels of TNF-α, IFN-γ, IL-6, and IL-1β were measured using corresponding ELISA kits according to the manufacturer’s instructions.

### 2.10 Western blotting analysis

Cells were collected from RAW 264.7 macrophages of the following groups: blank control, model control, positive control, BSP treated (500 μg/mL), and BSP-2 treated (500 μg/mL), washed with phosphate-buffered saline (PBS), detached using trypsin, and reseeded into fresh medium. Cells were then seeded into 96-well plates at a density of 1.5 × 10^5^ cells/mL and cultured for 12 h. Subsequently, 100 μL of lysis buffer was added to each well, and cells were lysed on ice for 30 min. After centrifugation (10,000 rpm, 10 min, 4 °C), the supernatants were collected, and protein concentrations were determined using a BCA protein assay kit and normalized accordingly.

A total of 20 μg of protein from each sample was separated by SDS-PAGE (stacking gel: 80 V; separating gel: 100 V) and then transferred onto a PVDF membrane at a constant voltage of 100 V for 90 min in an ice bath. The membrane was blocked with 5% skim milk at room temperature for 2 h, followed by incubation overnight at 4 °C with primary antibodies against COX-2, iNOS, p65, p-p65, IκBα, p-IκBα, and β-actin (all diluted 1:1000, except β-actin at 1:5000). After washing four times with TBST (5 min each), the membrane was incubated with a horseradish peroxidase (HRP)-conjugated secondary antibody (1:10,000) at room temperature for 1 h, washed again, and visualized using an ECL chemiluminescence kit. Images were captured with a gel imaging system (20).

Quantitative analysis was performed using ImageJ software. The relative expression levels of COX-2 and iNOS were normalized to β-actin. The phosphorylation ratios of IκBα and p65 were calculated by comparing the band intensities of p-IκBα to total IκBα and p-p65 to total p65, respectively. All experiments were performed with three independent biological replicates.

### 2.11 Statistical analysis

Statistical analysis was performed using SPSS 26.0 software. One-way analysis of variance (one-way ANOVA) was applied for comparisons among groups. Prior to ANOVA, the normality of data distribution was assessed using the Shapiro–Wilk test, and homogeneity of variances was verified by Levene’s test. Multiple comparisons were conducted with Tukey’s post-hoc test, and the significance level was set at α = 0.05. All experiments were carried out with six independent biological replicates (3 for immunoblotting), each containing three technical parallel samples. The sample size was determined based on common standards used in similar studies.

## 3 Results

### 3.1 Preparation of BSP-2

**Figure 1:**
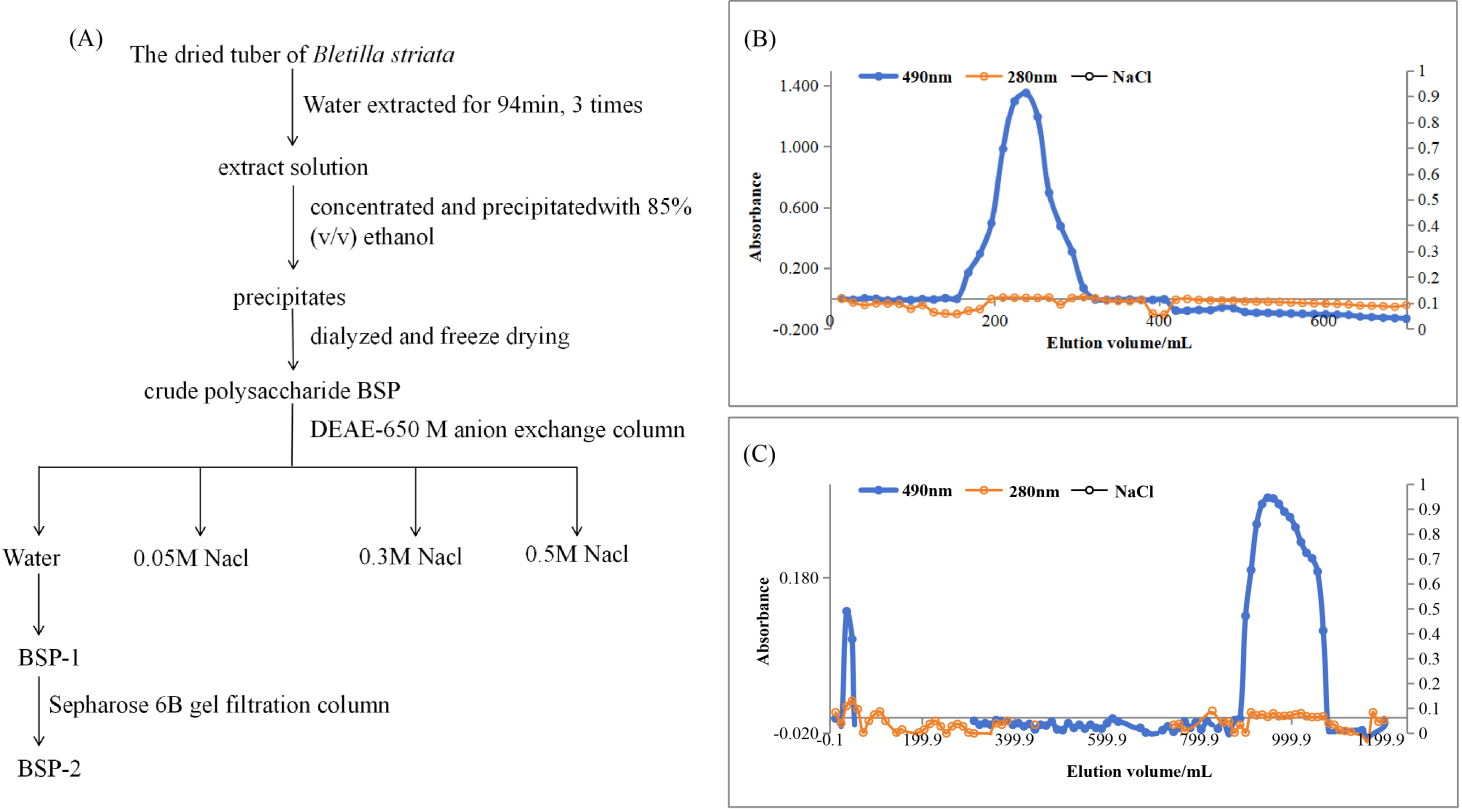
**(A)** Schematic diagram of the extraction and purification procedure of BSP-2; **(B)** Elution profile of BSP-2 on a DEAE-650M anion-exchange column; **(C)** Elution profile of BSP-2 on a Sepharose 6B gel-filtration column.

The yields of the purified fractions were 21.69 % for BSP-1 after DEAE-650M chromatography and 68.19 % for BSP-2 following Sepharose 6B column purification. BSP-2 was obtained as a water-soluble white powder. The extraction, purification procedure, and corresponding elution profiles are illustrated in **Figure 1**.

### 3.2 Molecular weight and homogeneity of BSP-2

**Figure 2:**
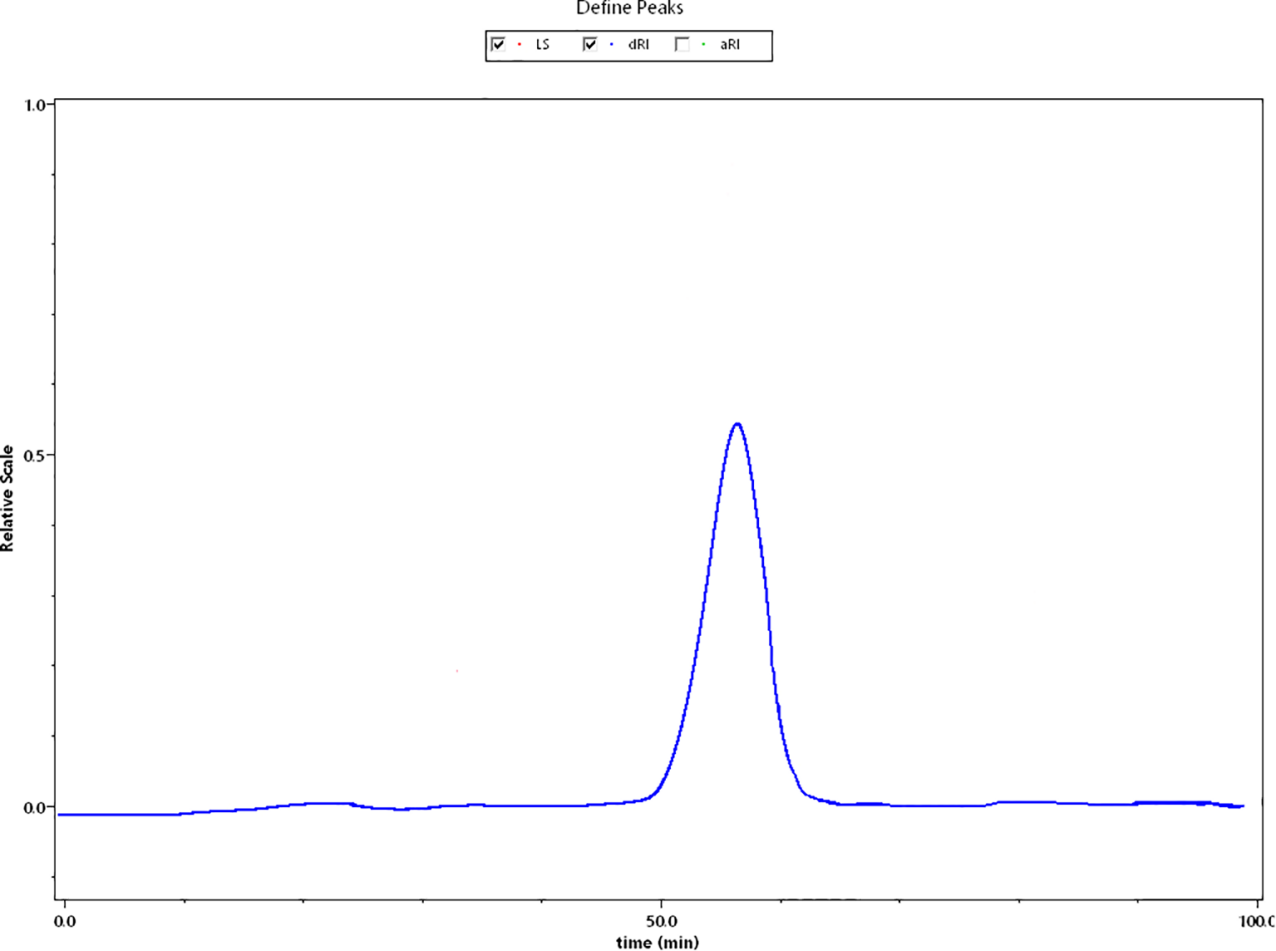
HPGPC chromatogram of BSP-2.

The HPGPC profile of BSP-2 displayed a single, sharp, and symmetrical peak (**Figure  2**). The molecular weight parameters were determined as follows: number-average molecular weight (M_n_) = 3.612 ± 0.13646 kDa; weight-average molecular weight (Mw) = 4.456 ± 0.18019 kDa; z-average molecular weight (Mz) = 6.364 ± 0.5518 kDa; peak molecular weight (Mp) = 2.808 ± 0.09041 kDa. The polydispersity index (Mw/M_n_) was 1.234 ± 0.22603.

### 3.3 Structural characterization of BSP-2

**Figure 3:**
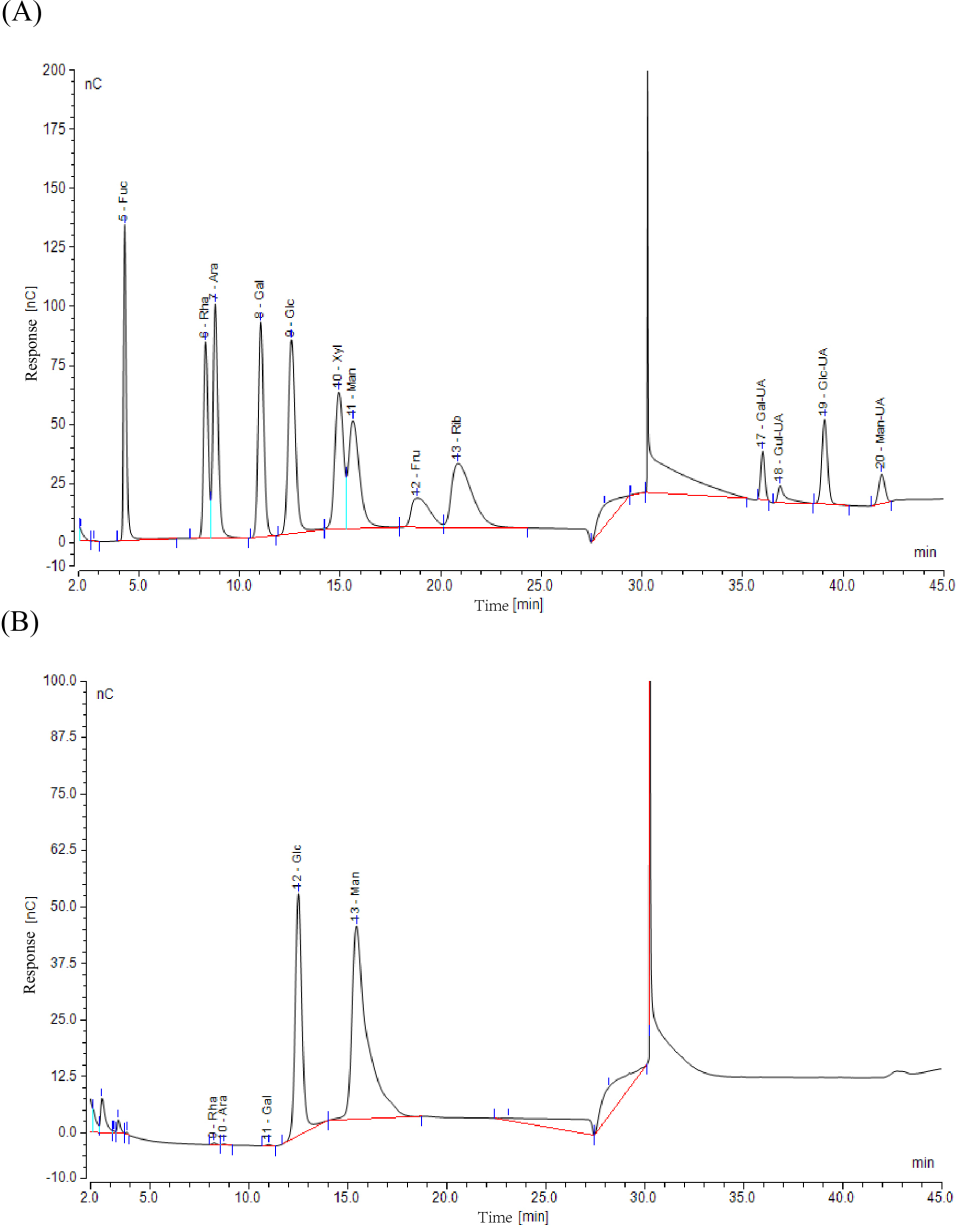
Chromatographic analysis of monosaccharide composition: **(A)** Chromatogram of monosaccharide standards; **(B)** Chromatogram of the sample’s monosaccharide composition.

Purity analysis revealed that BSP-2 contained 96.9 % total sugar and negligible amounts of protein. As shown in **Figure  3**, comparison with standard monosaccharide profiles indicated that BSP-2 is primarily composed of Man and Glc in an approximate ratio of 3:1, along with minor quantities of Gal, Ara, and Rha. No uronic acids were detected. Detailed results are presented in **Table 1**.

**Table 1:** Characteristics of BSP-2: molecular weight, chemical composition, and monosaccharide profile.

Polysaccharides	BSP-2	
Molecular weight (kDa)	Number-average molecular weight	3.612 ± 0.13646
	Weight average molecular weight	4.456 ± 0.18019
	Z-average molecular weight	6.364 ± 0.5518
	Peak molecular weight	2.808 ± 0.09041
Chemical composition (%)	Total carbohydrate	96.9%
	Glucuronic acid content	0
	Protein content	Trace
Monosaccharide composition (mol %)	Arabinose	0.0410
	Rhamnose	0.087
	Galactose	0.0498
	Glucose	12.0113
	Mannose	32.6824

kDa, kilodalton, a unit of molecular mass; mol%, mole percent, indicating the percentage of moles of a substance relative to the total moles in a mixture.

Methylation analysis indicated that the backbone of BSP-2 is predominantly composed of 4-Man (p) and 4-Glc (p) residues. The approximate 3:1 ratio suggests that the fraction is rich in mannose residues and possesses a highly branched glucoxylan structure. Furthermore, terminal-linked t-Man (p) and t-Glc (p) residues, as well as minor amounts of 3,4-Glc (p), 2,4-Glc (p), and 4,6-Glc (p) residues, were detected, implying that BSP-2 may represent a typical galactomannan containing a low proportion of glucogalactan. Detailed data are provided in **Table 2**.

**Table 2:** Structural connectivity of BSP-2.

Sample	Coupling means	Derivative name	Molecular weight of derivatives(MW)	Relative molar ratio(%)
BSP-2	t-Man(p)	1,5-di-O-acetyl-2,3,4,6-tetra-O-methyl mannitol	323	6.61
	t-Glc(p)	1,5-di-O-acetyl-2,3,4,6-tetra-O-methyl glucitol	323	3.30
	4-Man(p)	1,4,5-tri-O-acetyl-2,3,6-tri-O-methyl mannitol	351	62.81
	4-Glc(p)	1,4,5-tri-O-acetyl-2,3,6-tri-O-methyl glucitol	351	23.72
	3,4-Glc(p)	1,3,4,5-tetra-O-acetyl-2,6-di-O-methyl glucitol	379	1.11
	2,4-Glc(p)	1,2,4,5-tetra-O-acetyl-3,6-di-O-methyl glucitol	379	1.08
	4,6-Glc(p)	1,4,5,6-tetra-O-acetyl-2,3-di-O-methyl glucitol	379	1.37

t: indicates a terminal sugar residue; p: indicates a pyranose ring structure; Relative molar ratio (%): expressed as a percentage of the relative molar proportion, representing the molar contribution of the corresponding derivative in the sample.

**Figure 4:**
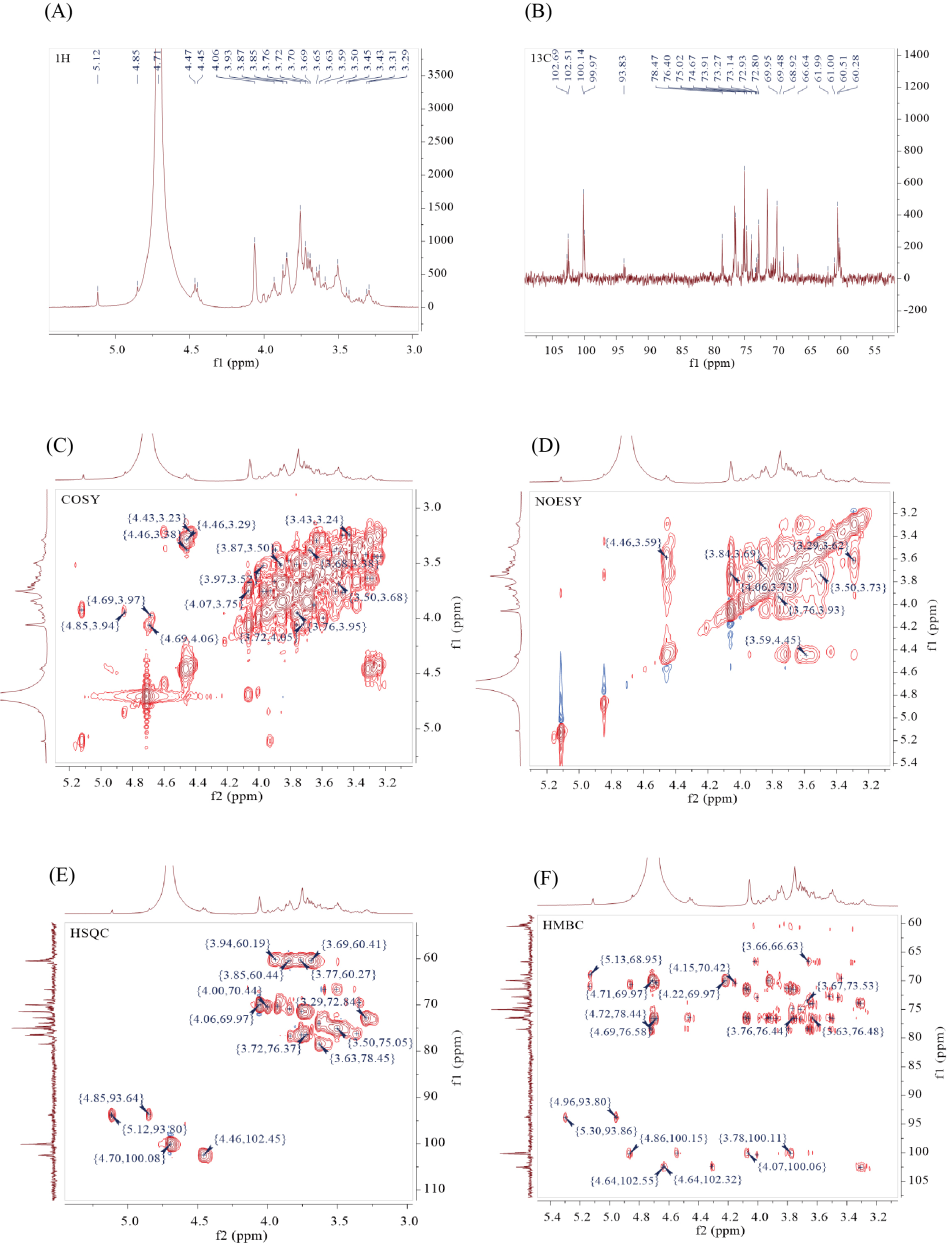
NMR characterization of BSP-2: **(A)** ¹H NMR spectrum, **(B)** ¹³C NMR spectrum, **(C)** COSY spectrum, **(D)** NOESY spectrum, **(E)** HSQC spectrum, and **(F)** HMBC spectrum.

As shown in **Figure 4A**, the ¹H NMR spectrum displayed proton signals of the polysaccharide mainly in the chemical shift range of 3–6 ppm. Signals for β-anomeric protons are generally observed at δ 4.3–4.8 ppm, while α-anomeric protons typically resonate at δ 4.8–5.8 ppm (21). The presence of multiple correlated anomeric proton signals (δ 4.3–5.4 ppm) with characteristic shifts at δ 5.12, 4.85, 4.47, and 4.45 ppm indicated that the sample likely contains different types of sugar residues. The majority of signals in the δ 3.1–4.2 ppm region corresponded to ring protons (H2–H6) of sugar residues, with considerable signal overlap. For structural elucidation, non-anomeric protons (H2–H6) were explicitly assigned (22, 23). The sample exhibited a strong signal around δ 4.71 ppm.

In the ¹³C NMR spectrum (**Figure 4B**), polysaccharide signals in the range of 95–110 ppm were attributed to anomeric carbons. The polysaccharide displayed characteristic anomeric carbon signals. Connectivity within the anomeric region was confirmed by ¹³C-¹H heteronuclear correlation, revealing potential terminal groups at the following shifts: δ 4.69/100.14, 4.47/102.51, 4.68/99.97, 5.12/93.83, 4.45/102.69, and 4.85/93.64 ppm. The identified sugar residues were systematically labeled A through F.

Based on preliminary identification results—including linkage information from methylation analysis, terminal signals, and comprehensive reports from relevant literature—combined with NMR evidence, the sugar residues were tentatively assigned as follows: A: →4)-β-D-Manp-(1→, B: →4)-β-D-Glcp-(1→, C: β-D-Manp-(1→, D: α-D-Glcp-(1→, F: →3,4)-α-D-Glcp-(1→. Accordingly, the ¹H and ¹³C chemical shifts of the sugar residues in the sample were sequentially assigned (24–28). The H1 proton signals of residues A and C overlapped with the solvent peak and are therefore not labeled in the ¹H NMR spectrum; all other signals are indicated (29, 30). Specific details are provided in **Table 3**.The COSY spectrum of BSP-2 (**Figure  4C**) identified correlation peaks between adjacent or geminal protons and diagonal peaks. The NOESY spectrum (**Figure  4D**) displayed relevant signals with corresponding cross-peaks annotated. The HSQC spectrum of the sample is presented in **Figure  4E**, and its HMBC spectrum is shown in **Figure  4F**, with key correlation signals indicated. Based on the NMR spectra of BSP-2, the signals of sugar residues were assigned as summarized in **Table 3**.

**Table 3:** Chemical shift assignment of BSP-2 (δ, ppm).

Code	Glycosyl residues	Chemical shifts(ppm)					
		H1/C1	H2/C2	H3/C3	H4/C4	H5/C5	H6/C6
A	→4)-β-D-Manp-(1→	4.69	4.06	3.74	3.75	3.5	3.51, 3.59
		100.14	69.97	71.25	76.5	74.5	66.65
B	→4)-β-D-Glcp-(1→	4.47	3.29	3.61	3.63	3.6	3.94, 3.8
		102.51	72.84	73.94	78.45	71.84	60.19
C	β-D-Manp-(1→	4.68	3.96	4.06	4.1	3.97	3.69, 3.62
		99.97	69.84	71.25	69.8	70.43	60.41
D	α-D-Glcp-(1→	5.12	3.92	3.44	3.6	3.23	3.77, 3.98
		93.83	69.44	73.94	72.5	73.25	60.27
E	→4,6)-β-D-Glcp-(1→	4.45	3.38	3.73	3.84	3.59	3.34, 3.44
		102.69	74.93	72.2	76.72	71	69.47
F	→3,4)-α-D-Glcp-(1→	4.85	3.94	3.72	3.58	3.61	3.85, 3.5
		93.64	70.27	76.37	78.47	72.01	60.44

Heteronuclear multiple-bond correlation (HMBC) spectroscopy was employed to determine and analyze the ¹³C and ¹H chemical shifts of various sugar residues. Key cross-peaks included δ 4.69/76.5 ppm (A-H1 and A-C4) and δ 4.69/69.47 ppm (E-C6). A distinct cross-peak at δ 4.47/76.5 ppm indicated a linkage between B-H1 of a hexosamine residue and A-C4, while the cross-peak at δ 4.68/76.72 ppm connected C-H1 with E-C4. Subsequent NOESY analysis revealed the sequence of residues, with cross-peaks appearing at δ 4.47/3.75 ppm (B-H1 and A-H4) and δ 4.47/3.63 ppm (B-H4 and B-H4). Additionally, a cross-peak at δ 4.45/3.63 ppm confirmed the interaction between E-H1 and B-H4. Residues D and F were excluded from the structural characterization due to their very low abundance.

**Figure 5:**
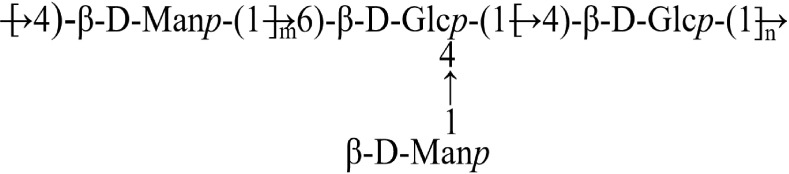
Putative structure of BSP-2.

Structural analysis integrating 1D/2D NMR and methylation data indicates that the polysaccharide backbone is primarily composed of →4)-β-D-Manp-(1→ and →4)-β-D-Glcp-(1→ linkages, with minor branches of →4,6)-β-D-Glcp-(1→. The side chains consist mainly of β-D-Manp-(1→ units attached at the O-4 position of the →4,6)-β-D-Glcp-(1→ residues. Based on all chemical and instrumental analytical methods applied for polysaccharide characterization, a proposed structure has been deduced, as illustrated in **Figure 5**.

### 3.4 Effects of BSP-2 on cell viability

**Figure 6:**
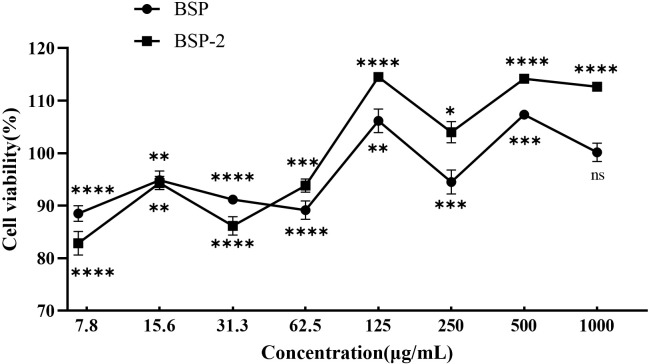
Effects of BSP and BSP-2 on the viability of RAW 264.7 macrophages.(n=6) *p < 0.05, **p < 0.01, ***p < 0.001, ****p < 0.0001 compared to the negative control (cell viability set as 100%); ns, not significant (*p* > 0.05).

**Figure 6** illustrates the effects of BSP and BSP-2 on the viability of RAW 264.7 cells. Within the concentration range of 7.8–62.5 μg/mL, both polysaccharides exhibited varying degrees of inhibitory effects on cell proliferation. At concentrations above 125 μg/mL, they promoted cell proliferation; however, this promoting effect was reduced at 250 and 1000 μg/mL.

### 3.5 Effects of BSP-2 on cytokine secretion

**Figure 7:**
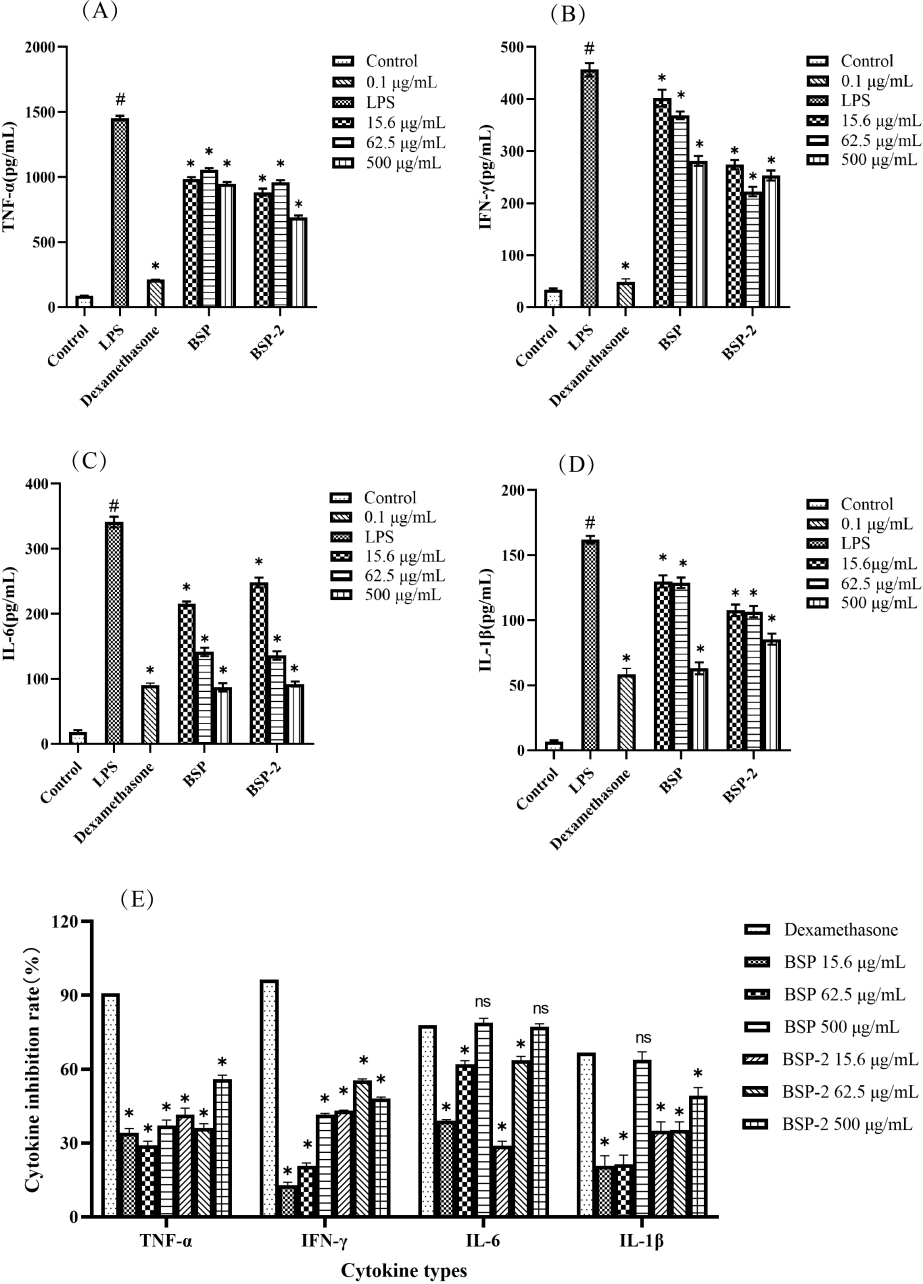
Effects of BSP and BSP-2 on the secretion of **(A)** TNF-α, **(B)** IFN-γ, **(C)** IL-6, and **(D)** IL-1β by RAW 264.7 macrophages. #*p* < 0.05 *vs*. the blank group; *p < 0.05 *vs*. the model group. **(E)** Inhibition rates of the positive control, BSP, and BSP-2 on TNF-α, IFN-γ, IL-6, and IL-1β. *p < 0.05 *vs*. the positive control group; ns, not significant (*p* > 0.05) *vs*. the positive control group.(n=6).

**Figures 7A–D** show the effects of BSP and BSP-2 on the secretion of cytokines (TNF-α, IFN-γ, IL-6, and IL-1β) by RAW 264.7 macrophages. Compared with the blank control group, the model group exhibited significantly elevated levels of TNF-α, IFN-γ, IL-6, and IL-1β (*p* < 0.05). Treatment with the positive control, BSP, or BSP-2 significantly reduced the secretion of these cytokines relative to the model group (*p* < 0.05). Notably, the inhibitory effects of BSP and BSP-2 on IL-6 secretion were dose-dependent.

**Figure 7E** compares the percentage inhibition of TNF-α, IFN-γ, IL-6, and IL-1β by the positive control, BSP, and BSP-2. At 500 μg/mL, BSP inhibited IL-6 and IL-1β by 78.83 % and 63.74 %, respectively, showing no significant difference compared to the positive control group (p > 0.05). Similarly, BSP-2 at 500 μg/mL inhibited IL-6 by 77.28 %, which was also not significantly different from the positive control (p > 0.05).

### 3.6 Western blotting analysis

**Figure 8:**
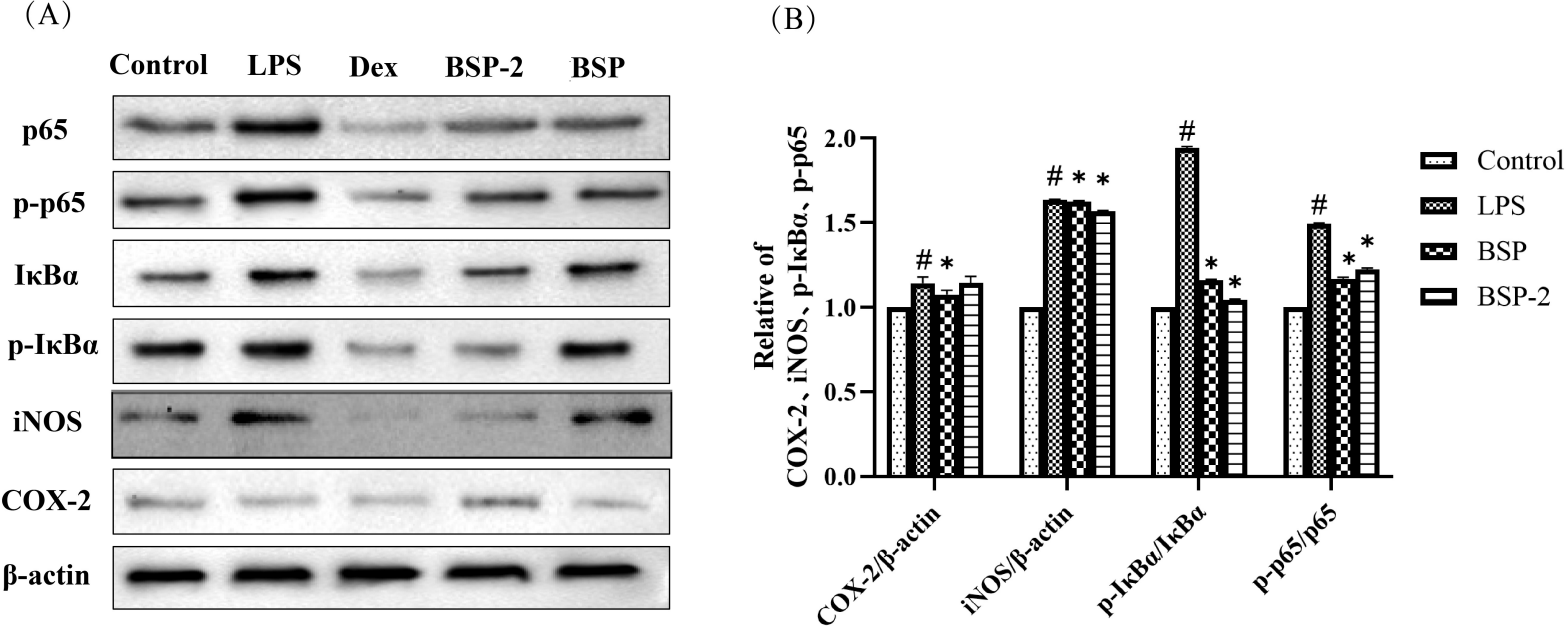
**(A)** Western blot analysis of p-p65, p65, p-IκBα, IκBα, iNOS, and COX-2 expression in RAW 264.7 macrophages. Lanes from left to right: blank group, model group, positive control group, BSP-2-treated group, and BSP-treated group. **(B)** Relative expression levels of COX-2, iNOS, p-p65/p65, and p-IκBα/IκBα. #*p* < 0.05 *vs*. the blank group; *p < 0.05 *vs*. the model group.(n=3).

**Figure 8A** illustrates the effects of high doses of BSP and BSP-2 on NF-κB pathway markers (iNOS, COX-2, p65, p-p65, IκBα, and p-IκBα). Compared to the blank group, the model group showed significantly elevated expression levels of p65, p-p65, IκBα, and p-IκBα (*p* < 0.05). In contrast, treatment with BSP and BSP-2 significantly reduced the expression levels of these proteins compared to the model group (*p* < 0.05).

LPS stimulation significantly increased the expression of iNOS protein compared to the blank group (*p* < 0.05). Relative to the model group, BSP-2 significantly downregulated iNOS expression (*p* < 0.05), whereas BSP exhibited a more pronounced inhibitory effect on COX-2 (*p* < 0.05).

## 4 Conclusion

The structure of polysaccharides is closely linked to their bioactivity. Different extraction and purification methods yield BSPs with varying molecular weights and biological activities. Hot-water extraction typically produces high-molecular-weight fractions (282.91–402.17 kDa), while alkaline extraction and ultrasound-assisted methods often yield lower-molecular-weight fractions (195.83–230.63 kDa) (31–34). Purification of crude BSP commonly employs ion-exchange chromatography (e.g., DEAE-cellulose, DEAE-cellulose-52, DEAE Sepharose Fast Flow) to separate neutral from acidic polysaccharides and remove impurities such as proteins and pigments, followed by gel-filtration chromatography (e.g., Sephadex G-200, Bio-Gel P300, Sephadex L columns) to fractionate BSPs by molecular weight (35).

In this study, BSP was extracted using hot water, preliminarily fractionated on a DEAE-650M anion-exchange column to obtain the neutral polysaccharide fraction BSP-1, and further purified by Sepharose 6B gel-filtration chromatography to yield the homogeneous fraction BSP-2. Structural characterization revealed that BSP-2 had a purity of 96.9 % and a molecular weight of 4.456 kDa. Its monosaccharide composition included Man, Glc, Rha, Gal, and Ara in a molar ratio of 32.6824 : 12.0113 : 0.0870 : 0.0498 : 0.0410. Both the molecular weight and monosaccharide molar ratio differ substantially from previously reported BSPs, indicating that BSP-2 is a previously uncharacterized, high-purity, low-molecular-weight neutral polysaccharide from *Bletilla striata*.

Previous studies have demonstrated the immunomodulatory activity of BSPs. In the present study, we examined the effects of BSP-2 on cell viability, secretion of inflammatory cytokines, and expression of NF-κB pathway-related proteins in RAW 264.7 macrophages, thereby confirming that BSP-2, as a low-molecular-weight polysaccharide, possesses anti-inflammatory and immunoregulatory properties.

The impact of two polysaccharide fractions, crude BSP and purified BSP-2, on the viability of RAW 264.7 macrophages was evaluated across a concentration gradient of 7.8–1000 µg/mL. At low to medium concentrations (7.8–62.5 µg/mL), both fractions exhibited varying degrees of inhibitory effects on cell viability, whereas at medium to high concentrations (125–1000 µg/mL), they promoted cell proliferation. This biphasic effect resembles the proliferative activity reported for a polysaccharide from the *Pyrus sinkiangensis Yu* (36). The underlying mechanism may involve mild induction of apoptosis or inhibition of proliferation signaling at lower concentrations, while at higher concentrations, the polysaccharides may act as nutrients that enhance cellular metabolism and subsequently promote proliferation.

Within this concentration range, 15.6 and 62.5 µg/mL (low and medium doses, respectively) slightly inhibited RAW 264.7 macrophages, although cell viability remained around 90 %. In contrast, 500 µg/mL (high dose) significantly promoted proliferation, while further increasing the concentration to 1000 µg/mL reduced this promoting effect. Based on these observations, 15.6, 62.5, and 500 µg/mL were selected as safe low, medium, and high doses, respectively, for subsequent cellular experiments.

Both BSP and BSP-2 significantly reduced the excessive secretion of TNF-α, IFN-γ, IL-6, and IL-1β in LPS-induced RAW 264.7 macrophages. The inhibitory effect of high doses of BSP and BSP-2 on IL-6 showed no significant difference compared with that of the positive control (*p *< 0.05) and exhibited a dose-dependent trend. Western blot results indicated that although both BSP and BSP-2 suppressed NF-κB activation markers (p-p65, p-IκBα), they displayed compound-specific activities: BSP-2 reduced iNOS expression, whereas BSP did not show a clear inhibitory effect on iNOS but lowered COX-2 levels. It is thus suggested that the anti-inflammatory activities of BSP and BSP-2 may be mediated through the inhibition of NF-κB pathway-related proteins.

In conclusion, our study identifies BSP-2 as a novel, low-molecular-weight polysaccharide from *Bletilla striata* with significant immunomodulatory properties. The data collectively demonstrate that BSP-2 can modulate macrophage function by promoting proliferation at higher concentrations, suppressing pro-inflammatory cytokine secretion, and inhibiting the NF-κB signaling pathway. These findings align with the reported bioactivities of polysaccharides and substantiate the potential of BSP-2 as an adjuvant therapeutic agent or a functional food ingredient for immunomodulation.

It should be noted that a limitation of this study is the lack of direct evidence for p65 nuclear translocation, a central event in NF-κB activation. Future work employing techniques such as immunofluorescence is warranted to fully delineate the mechanistic details, thereby strengthening the foundation for developing *B. striata* polysaccharides as targeted immunomodulatory agents.

## Data Availability

The datasets presented in this study can be found in online repositories. The names of the repository/repositories and accession number(s) can be found below: The datasets generated and analyzed during the current study are available in the Figshare repository, “Structural Identification and Anti-inflammatory Activity Data of Bletilla striata Polysaccharide”, with the DOI: 10.6084/m9.figshare.30399400.
